# Intraoperative Microelectrode Recordings in Substantia Nigra Pars Reticulata in Anesthetized Rats

**DOI:** 10.3389/fnins.2020.00367

**Published:** 2020-04-29

**Authors:** Hanyan Li, George C. McConnell

**Affiliations:** Department of Biomedical Engineering, Stevens Institute of Technology, Hoboken, NJ, United States

**Keywords:** deep brain stimulation, Parkinson’s disease, intraoperative microelectrode recordings, action potentials, Substantia Nigra pars reticulata

## Abstract

The Substantia Nigra pars reticulata (SNr) is a promising target for deep brain stimulation (DBS) to treat the gait and postural disturbances in Parkinson’s disease (PD). Positioning the DBS electrode within the SNr is critical for the development of preclinical models of SNr DBS to investigate underlying mechanisms. However, a complete characterization of intraoperative microelectrode recordings in the SNr to guide DBS electrode placement is lacking. In this study, we recorded extracellular single-unit activity in anesthetized rats at multiple locations in the medial SNr (mSNr), lateral SNr (lSNr), and the Ventral Tegmental Area (VTA). Immunohistochemistry and fluorescently dyed electrodes were used to map neural recordings to neuroanatomy. Neural recordings were analyzed in the time domain (i.e., firing rate, interspike interval (ISI) correlation, ISI variance, regularity, spike amplitude, signal-to-noise ratio, half-width, asymmetry, and latency) and the frequency domain (i.e., spectral power in frequency bands of interest). Spike amplitude decreased and ISI correlation increased in the mSNr versus the lSNr. Spike amplitude, signal-to-noise ratio, and ISI correlation increased in the VTA versus the mSNr. ISI correlation increased in the VTA versus the lSNr. Spectral power in the VTA increased versus: (1) the mSNr in the 20–30 Hz band and (2) the lSNr in the 20–40 Hz band. No significant differences were observed between structures for any other feature analyzed. Our results shed light on the heterogeneity of the SNr and suggest electrophysiological features to promote precise targeting of SNr subregions during stereotaxic surgery.

## Introduction

Deep brain stimulation (DBS) is an effective treatment for tremor, rigidity, and bradykinesia in Parkinson’s disease (PD). Although these distal symptoms are reliably treated by DBS and dopaminergic medication, the axial symptoms of gait and postural disturbances continue to worsen 5 years after implant (St George et al., 2010). The gait and postural disturbances are difficult to treat by either medication (Curtze et al., 2016) or DBS at the Subthalamic Nucleus (STN) (St George et al., 2010). Substantia Nigra pars reticulata (SNr) is a promising DBS target to treat the gait and postural disturbances in PD (Chastan et al., 2009; Weiss et al., 2011, 2013; Brosius et al., 2015; Scholten et al., 2017; Valldeoriola et al., 2019). Location of the DBS electrode within the SNr may play a crucial role in effective treatment, but the neural mechanisms for the location dependence of SNr DBS are not clear. Studies in rats (McConnell and Grill, 2013), cats (Takakusaki et al., 2003), and humans (Scholten et al., 2017) suggest that stimulation at lateral SNr (lSNr) sites is less effective at treating the gait disturbances of PD compared to stimulation at medial SNr (mSNr) sites.

The lateralization of SNr DBS may stem from the anatomical and functional heterogeneity of the SNr. The mSNr and lSNr receive different projections from the sensorimotor and limbic striatum, respectively (Deniau et al., 1996; Mailly et al., 2001). GABAergic neurons of mSNr and lSNr have a differential change of firing activity in PD (Wang et al., 2010). Ablation of the mSNr results in contralateral turning behavior, while ablation of the lSNr results in ipsilateral turning behavior in rats (Franklin and Wolfe, 1987).

Intraoperative microelectrode recordings (MERs) are commonly used to verify and refine targeting of DBS electrode placement during STN DBS surgery for PD. MERs employ extracellular recordings of action potentials along preplanned trajectories (Benazzouz et al., 2002) to minimize misplacement of the DBS electrode, which may result in unwanted side effects (Chan et al., 2009; Valsky et al., 2017). We hypothesized that the waveform shape and neuronal firing patterns of spikes detected from MERs within the SNr differ depending on the location of the electrode within SNr subregions. In addition to characterizing MERs in the mSNr and the lSNr, we investigated the capability of MERs to define structures surrounding the SNr including the Substantia Nigra pars compacta (SNc; dorsal to SNr) and Ventral Tegmental Area (VTA; medial to SNr). We recorded the subregions and surroundings of the SNr, namely the mSNr, the lSNr, the SNc and the VTA, in an anesthetized healthy rat model. MERs in anesthetized healthy rats is consistent with the future application of SNr DBS or SNr electrophysiology in parkinsonian rats by chronically implanting a cannula for 6-hydroxydopamine (6-OHDA) infusion to later render the rats parkinsonian (McConnell et al., 2016). Each recording site was confirmed postmortem by identification of the electrode track and immunohistochemistry to compare electrophysiological features of action potentials recorded in the mSNr, the lSNr, and the VTA.

## Methods

### Electrode Preparation

Each tungsten microelectrode (MicroProbes; diameter = 75 μm; impedance = 0.5 MΩ) was oriented vertically, dipped 10 times into DiI (Molecular Probes, 50 mg/ml solution concentration) and allowed to dry in air 5 s between dips (DiCarlo et al., 1996). Following the dip-coating procedure, each electrode was inspected under a microscope to confirm that it was undamaged and uniformly coated to the tip of the electrode.

### Surgery

All animal care and experimental procedures were approved by the Stevens Institute of Technology Institutional Animal Care and Use Committee. Stereotaxic (Stoelting) surgery was conducted in anesthetized Long-Evans female rats (250–300 g) (*n* = 13). Anesthesia was induced at 7% Sevoflurane (Piramal Petrem) and maintained under 4% Sevoflurane. A craniotomy was made over the SNr and the electrode was lowered into the brain at the coordinates: anterior/posterior: 5.5 mm; medial/lateral (ML) 1.5 mm from the midline for mSNr and ML 2.3 mm from the midline for lSNr. Data were recorded from 6.0 to 9.0 mm from the cortical surface in 0.1 mm steps for 30 s at each location for a total of 31 recordings per insertion track. One mSNr and one lSNr insertion track was made in each brain hemisphere. The recorded anatomical structure was confirmed by postmortem histology. A Grapevine Scout neural recording system (Ripple) was used to record the raw data, which was sampled at 30 kHz.

### Immunohistochemistry

Immediately following the surgery, rats were intracardially perfused with Phosphate-buffered saline prewash followed by 10% formalin. Following perfusion, brains were extracted and fixed in 10% formalin overnight, followed by 30% sucrose solution until the brain sank to the bottom. The left side of the cortical surface was marked with green dye (Triangle Biomedical Science) to determine the left and right hemispheres under bright field microscopy. The brain samples were cryoprotected with Optimal Cutting Temperature (O.C.T.) compound and stored in −80°C overnight. Samples were then sectioned using a cryostat (Thermo Scientific CryoStar NX50) at −23°C (thickness = 40 μm). Tissue sections containing the SNr were immunostained for Tyrosine Hydroxylase (TH) (Sigma-Aldrich) for dopaminergic neurons and cover-slipped using DAPI Fluoromount-G (Southern- Biotech) for all cell nuclei. TH immunostaining confirmed the microelectrode tip location by visualizing the SNc to aid in identification of the SNr (McConnell et al., 2012, 2016; So et al., 2017). Only recordings of tracks confirmed to pass through the SNr and/or the VTA were further analyzed.

### Data Analysis

#### Spike Sorting

Action potentials were obtained by 4th order Butterworth bandpass filtering the raw neural data from 300–5000 Hz and sorted using the Wave_clus toolbox (Quiroga et al., 2004) in MATLAB (Mathworks). The detection threshold was set within the background noise (threshold type: negative, minimal threshold for detection STD = 5, maximal threshold for detection STD = 50, minimal size of cluster = 60, sampling rate = 30 kHz) in order to include the background noise as one sorted cluster and facilitate visualization of a spike cluster. Background noise was defined as the amplitude of the noise cluster after spike sorting. While Principal Component Analysis (PCA) is more generally applied for spike sorting, wavelet transform can outperform PCA in some datasets (Pavlov et al., 2007). Therefore, wavelet transform was used as the spike sorting method to identify spikes for feature extraction.

#### Feature Extraction

**FIGURE 1:**
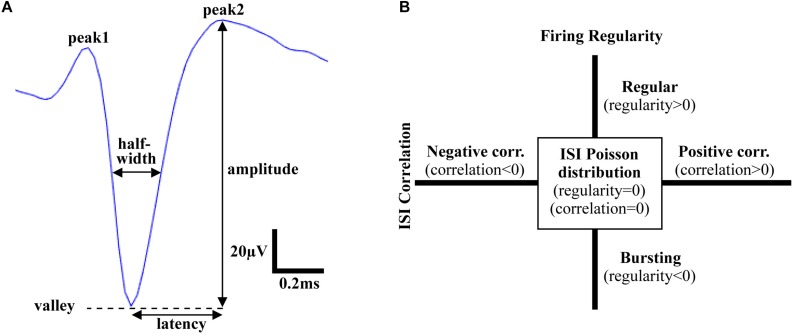
Time domain features of action potentials. **(A)** Waveform shape features. Amplitude: voltage from valley to peak; Latency: time from valley to the 2nd peak; Half-width: width at half amplitude; Asymmetry: ratio of amplitude of the 1st peak and the 2nd peak. **(B)** Firing pattern features. Firing regularity: the logarithm of the shape factor κ of the gamma distribution fitted to the interspike intervals (ISIs). A spike train was considered Poisson random if its ISIs were exponentially distributed (κ = 1 or log κ = 0), whereas it was considered regular if log κ > 0 or bursting if log κ < 0; ISI correlation: the Spearman’s rank-order correlation calculated for sequentially paired ISIs indicated whether consecutive ISIs were positively correlated or negatively correlated.

Following spike sorting, single-unit activity was analyzed using custom MATLAB software to extract features in both the time and frequency domains. Action potentials recorded in the VTA, the mSNr, and the lSNr, based on histology, were analyzed for feature extraction. In the time domain, firing temporal features and waveform shape features were investigated. Firing temporal features included: (1) firing rate – number of spikes per second [Hz]; (2) ISI correlation – the Spearman’s rank-order correlation computed for sequentially paired ISIs indicates whether consecutive ISIs are positively correlated or negatively correlated; (3) ISI variance – the variance of ISIs; (4) firing regularity – logarithm of the shape factor of the gamma distribution fitted to the ISIs resulting in classification of spike trains as Poisson random if its ISIs are exponentially distributed (i.e., regularity = 0), regular (i.e., regularity >0), or bursting (i.e., regularity <0) (Mochizuki et al., 2016; Figure 1B). Waveform shape features included: (5) spike amplitude - measured from the negative peak to the positive peak of the spike waveform [μV]; (6) signal-to-noise ratio – spike amplitude divided by background noise amplitude; (7) half-width – width when amplitude equals to half of the amplitude [ms]; (8) asymmetry – the ratio of the amplitude of 2nd positive peak and the 1st positive peak; (9) latency – time from negative peak to the 2nd positive peak [ms] (Figure 1A). For each electrode track, density was calculated as percentage of recording locations with spiking activity compared with the total number of recording locations [%]. In the frequency domain, the power of spike trains was evaluated by Chronux with sampling rate = 30 kHz, win = 5 s, tapers = [3 5], pad = 0, and frequency calculation band = [0 200] (Mitra and Pesaran, 1999). Band power was calculated as the sum of the spectral power over frequencies in the bands of interest: delta (1.5–4 Hz), theta (4–10 Hz), low beta (10–20 Hz), high beta (20–30 Hz), Gamma (30–40 Hz, 40–50 Hz, 50–60 Hz, 60–70 Hz, and 70–80 Hz).

#### Feature Comparison

Time domain: After each time domain feature was extracted, firing temporal features (firing rate, amplitude, ISI correlation, ISI variance, firing regularity) and waveform shape features (amplitude, signal-to-noise ratio, half-width, asymmetry, latency) from the mSNr, the lSNr and the VTA were compared.

Frequency domain: After power spectrum at each depth was calculated, power of each frequency was calculated and compared between the mSNr, the lSNr and the VTA. Contiguous frequencies with statistically significant differences in power were considered as a frequency band of interest.

#### Statistical Analysis

Statistical inferences were made between differing conditions using one-way ANOVA. When we found a significant factor, we performed the Fisher’s protected least significant difference (PLSD) *post hoc* test to identify pairwise differences. Student’s *t*-test were used where indicated. All results are presented as the mean ± SEM and were considered significant at *p* < 0.05.

## Results

### Histology

**FIGURE 2:**
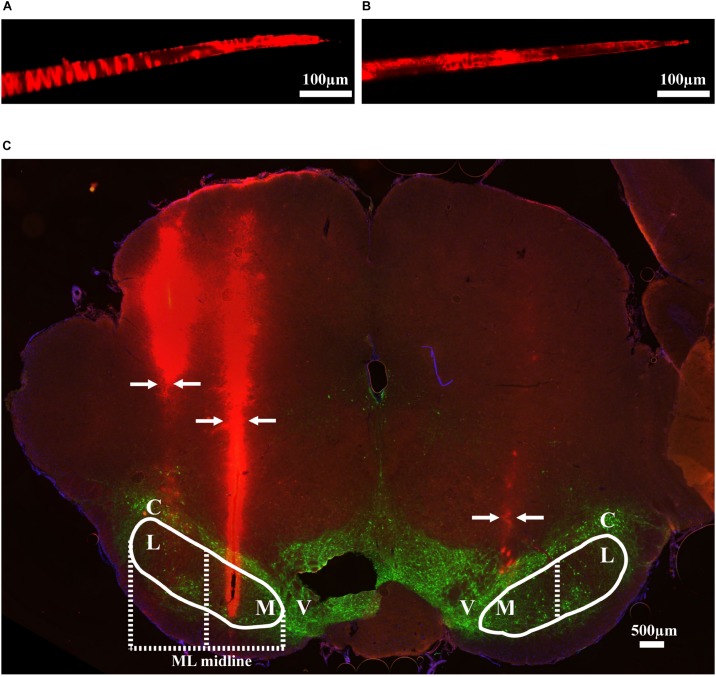
Methods used to validate electrode location. **(A)** An example of DiI coated electrode before brain insertion. **(B)** The same electrode after brain insertion. **(C)** Coronal brain section showing DiI coated electrodes in mSNr and lSNr. Red indicates electrode tracks coated with DiI; green indicates dopaminergic neurons in SNc and VTA stained by anti-tyrosine hydroxylase. Borders of SNr are indicated by the white solid line. mSNr and lSNr (separated by dashed white line) were defined by evenly dividing the SNr in the medial/lateral direction. Arrows indicate locations of the electrode tracks. Abbreviations: M, medial Substantia Nigra pars reticulata (mSNr); L, lateral Substantia Nigra pars reticulata (lSNr); C, Substantia Nigra pars compacta (SNc); V, ventral tegmental area (VTA).

**FIGURE 3:**
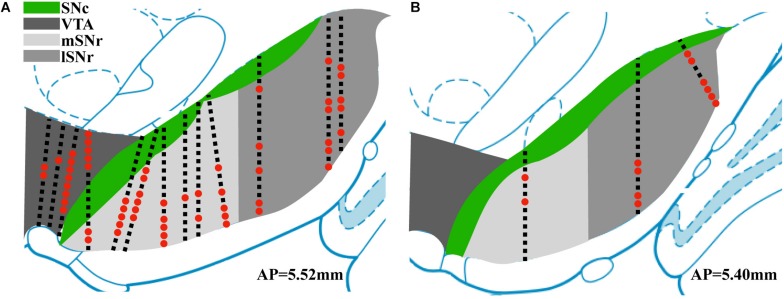
Summary of position of electrode tracks based on histology. **(A)** Electrode tracks in coronal section corresponding to AP 5.52 mm (Paxinos and Watson, 2007). **(B)** Electrode tracks in coronal section corresponding to AP 5.40 mm. Black squares indicate recording sites with no sorted single units. Red dots indicate recording sites with sorted single units.

Thirteen rats were recorded with 28 electrode insertions spanning the mSNr, the lSNr, and the VTA. Six insertions using bare electrodes confirmed that the quality of recordings was not altered by the DiI coating (data not shown). Twenty two tracks were made by DiI coated electrodes. DiI remained intact after insertion (Figures 2A,B). Sixteen out of the 22 DiI coated electrode tracks could be visualized by fluorescence microscopy. Dopaminergic neurons in the SNc and the VTA were visualized by TH immunohistochemistry. The dorsal border of the SNr was defined by an absence of TH staining and immediately ventral to the SNc. The midline in the medial/lateral direction within SNr was defined as the border line of the mSNr and the lSNr (Figure 2C). Out of 16 tracks, 8 tracks passed through mSNr, 5 tracks passed through the lSNr, and 4 tracks passed through the VTA (Figure 3A). One out of the 4 tracks that passed through the VTA also passed through the mSNr (Figure 3A). The mSNr dorsal border was approximately 6.4 mm from the cortical surface according to histology compared to 8.0 mm according to the stereotaxic atlas (Paxinos and Watson, 2007). The lSNr border was approximately 6.1 mm from the cortical surface according to histology compared to 7.3 mm according to the stereotaxic atlas (Paxinos and Watson, 2007).

### Spike Sorting

Neural recordings from electrode tracks confirmed by histology were further analyzed. The total number of recordings with histological confirmation was 496 (Figure 3). Following spike sorting, the total recordings with isolated single-unit activity was 72 with the remainder of recordings containing multi-unit activity and/or background noise. Fluorescent images were overlayed with the corresponding atlas panel (Paxinos and Watson, 2007), and all recording sites with single-unit activity were marked (Figure 3). Thirty out of 72 recording sites were in the mSNr, 26 out of 72 recording sites were in the lSNr, and 16 out of 72 recording sites were in the VTA. Single-unit activity was observed only in VTA, mSNr and lSNr; no spikes were detected dorsal or ventral to the VTA, dorsal to the SNc, or within the SNc.

### Feature Comparison

#### VTA vs. mSNr

**FIGURE 4:**
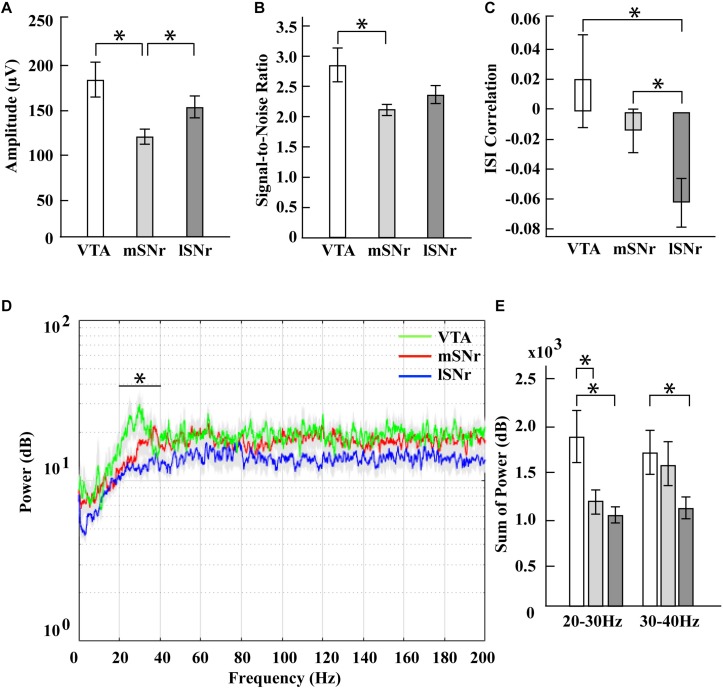
Electrophysiological results in mSNr, lSNr and VTA. **(A)** Spike amplitudes in mSNr, lSNr and VTA. **(B)** Signal-to-noise ratio in mSNr, lSNr and VTA. **(C)** ISI correlation in mSNr, lSNr and VTA. **(D)** Spectral power in mSNr, lSNr and VTA. Mean (colored lines) ± SEM (gray filled area surrounding colored lines) are shown. **(E)** Sum of power in 20–30 Hz and 30–40 Hz frequency bands. Mean ± SEM (SEM indicated by gray filled area surrounding lines) are shown. Features that showed statistically significant differences are indicated with *p < 0.05.

In the time domain, the significantly different features between VTA and mSNr were spike amplitude, signal-to-noise ratio (Figures 4A,B). Amplitude was significantly greater in the VTA compared to the mSNr (*p* = 0.0007) (Figure 4A). Signal-to-noise ratio was significantly greater in the VTA compared to the mSNr (*p* = 0.0028) (Figure 4B). In the frequency domain, the 20–30 Hz band was significantly decreased in the mSNr compared to the VTA (*p* = 0.0197) (Figures 4D,E). Thus, the VTA and the mSNr differed in waveform shape and neuronal firing pattern. No significant differences were observed between the VTA and the mSNr for any other feature analyzed (Supplementary Table S1).

#### VTA vs. lSNr

In the time domain, the significantly different feature between the VTA and the lSNr was ISI correlation (Figures 4A,C). The ISI correlation was positive in VTA but negative in lSNr (*p* = 0.0156) (Figure 4C). In the frequency domain, the 20–40 Hz band was significantly decreased in the lSNr compared to the VTA (*p* = 0.0118) (Figures 4D,E). Thus, the VTA and lSNr differed only in measures of neuronal firing pattern, with no significant differences were observed between the VTA and the lSNr for any other feature analyzed (Supplementary Table S1).

#### mSNr vs. lSNr

In the time domain, the significantly different features between the mSNr and the lSNr were spike amplitude and ISI correlation (Figures 4A,C). The spike amplitude in the mSNr decreased compared to the lSNr (*p* = 0.0224) (Figure 4A). The ISI correlation of mSNr increased compared to the lSNr (*p* = 0.0338) (Figure 4C). In the frequency domain, there were no significant differences between brain regions in any continuous frequency band (Figures 4D,E and Supplementary Table S1). Thus, the mSNr and the lSNr differed only in the time domain, for both waveform shape and neuronal firing pattern features. No significant differences were observed between the mSNr and lSNr for any other feature analyzed (Supplementary Table S1).

## Discussion

SNr DBS is a promising approach to treat the gait and postural disturbances of PD, but the neural basis is not clear. The position of the electrode within the SNr appears to play a role in effectively treating gait regularity in PD (McConnell and Grill, 2013; Scholten et al., 2017). In this study, we characterized intraoperative MERs of the SNr as an approach to improve the accuracy and precision of the stereotaxic placement of SNr DBS electrodes to study the underlying neural mechanisms. We characterized MERs across the SNr spanning medial and lateral subregions to compare both temporal and spectral features in the medial and lateral SNr, and the nearby VTA. Fluorescently labeled microelectrodes were used to map neuroanatomical position to MERs. Our results suggest that spike amplitude, signal-to-noise ratio and firing pattern differ depending on brain regions. These electrophysiological features may be useful markers to guide either SNr recording or stimulation microelectrode array placement in healthy rats, which could later be rendered parkinsonian via infusion of 6-OHDA in the medial forebrain bundle (Dorval and Grill, 2014). In this way, our results could improve the reliability of a parkinsonian rat model of SNr DBS for behavioral studies to investigate stimulation parameters and underlying neural mechanisms.

The location dependence of SNr DBS on the efficacy of treating gait may be attributed to several factors. Connections with Pedunculopontine Nucleus (PPN) of the brainstem suggest that SNr DBS modulates the descending network linking the SNr to the PPN (Pahapill and Lozano, 2000), which support the involvement of the SNr in gait and postural disturbances of PD. Also, it is possible that SNr DBS may increase SNc neuronal activity, because SNc is dorsal to, and interdigitated among the SNr. If so, high frequency stimulation induced excitation of SNc axons could induce dopaminergic release in the striatum and thereby improve PD symptoms (McIntyre et al., 2004; Zheng et al., 2011). In addition to the effects of SNr DBS location, frequency is another parameter that warrants more careful attention regarding mechanisms. Low stimulation SNr DBS (63 Hz) combined with high frequency STN DBS (130 Hz) was reported to improve freezing of gait in humans with PD (Valldeoriola et al., 2019). The mechanisms for these observed location and frequency effects of SNr DBS warrant further study in animal models of PD.

Although PPN is another promising DBS target for gait and postural disturbances in PD, PPN is a small midbrain region in comparison to the spatial extent of the stimulation effect produced by the microelectrode (Hamani et al., 2011). The lack of clarity of PPN contributes to the difficulty in targeting and determining the exact localization of the electrodes. It is likely that DBS in the PPN region affects neighbor structures. In humans the PPN overlaps with the posterior part of the Substantia Nigra (SN), so that it is presumably impossible to constrain stimulation to the PPN without also altering the SN. Hence, the observed effects on discrimination performance may at least to some degree stem from a modulation of activity in the SN (Strumpf et al., 2016). Because of the location, PPN DBS poses additional risk compared to other DBS surgeries (Welter et al., 2015). Stimulation-related adverse events during LFS PPN DSB include paresthesia, pain and temperature sensation, and some patients develop oscillopsia (a visual disturbance) during LFS PPN DBS (Fraix et al., 2010; Moro et al., 2010). One study reported that two out of six patients that received PPN DBS developed several adverse effects (Welter et al., 2015). For these reasons, there is growing interest in alternative DBS targets for gait and postural disturbances including the SNr.

Our results highlight the increased accuracy for electrode placement when using intraoperative MERs compared to stereotaxic coordinates from a rat brain atlas to localize deep brain structures. Previous studies of STN DBS in rodents reported a discrepancy between the dorsal/ventral coordinate predicted by MERs compared to the stereotaxic atlas (Gradinaru et al., 2009; McConnell et al., 2012, 2016). Here, the location of mSNr and lSNr according to histology and MERs was approximately 0.5–1.0 mm dorsal to the stereotaxic atlas coordinates. Thus, MERs may be advantageous for targeting compared to using a stereotaxic atlas alone. Future stereotaxic surgeries may consider adding intraoperative recordings in tandem with the traditional atlas to target deep brain structures with higher accuracy. It is common that during stereotaxic surgeries edema occurs due to drilling, which could contribute to the decreased accuracy the dorsal/ventral stereotaxic coordinates. The maximum swelling or denting of the cortex we observed was approximately 0.5 mm, which could result in missed targeting of a deep brain structure in the absence of MERs.

Our rationale for recording single-unit activity was to confine the measurement of extracellular potentials to the immediate vicinity of the microelectrode given the small dimensions of the SNr subregions in the rat. One limitation of single-unit recordings, however, is that sorted spikes are not observed at all depths along an electrode track. In some instances, single-unit activity was observed at only one recording site along the dorsal-ventral direction of an insertion track and the majority of the recordings we collected were excluded from this study due to an absence of sorted spikes. Local field potential (LFPs) to target SNr and its subregions could provide an alternative measure or additional measure to spiking activity relevant to SNr position (Li and McConnell, 2019). Unlike action potentials in SNr, LFPs can be measured at every recording site and thereby provide a more consistent measure but at a lower spatial resolution compared to single-unit recordings. Because a complete analysis of LFPs was beyond the scope of this study, we are at this time unable to correlate our histological results to features of LFPs. An additional limitation was the use of anesthetized healthy rats and not awake or parkinsonian rats. The 6-OHDA lesion model is the most commonly used pre-clinical parkinsonian rat model to investigate PD. The procedure to study DBS in 6-OHDA rats typically is to implant the electrode and cannula in anesthetized healthy rat brain (McConnell et al., 2016). Following a period of baseline healthy recordings, the cannula is then used to infuse 6-OHDA. In this way, longitudinal studies of DBS are possible with healthy and parkinsonian behavior and neural recordings from the same animal. Another limitation is that although each electrode was dye coated before surgery, with the same amount times of dipping and drying, only 16 out of 22 tracks were able to be visualized from histology. It is possible that the brain sections containing the electrode tracks were not saved during the sectioning procedure or torn during the immunohistochemistry protocol.

No spiking activity dorsal or ventral to the SNr suggests that it is feasible to use intraoperative recordings to delineate the dorsal and ventral borders of the SNr when using anesthetized healthy rat model. Electrophysiological features that showed significant differences between mSNr and VTA in the time domain were amplitude and signal-to-noise ratio. ISI correlation in VTA was positive while it was negative in both the mSNr and the lSNr. More specifically, the consecutive ISIs in VTA were positively correlated but the ISIs in mSNr and lSNr were negatively correlated. This suggests that VTA has a different firing pattern than either subregion of SNr. When targeting SNr, amplitude, signal-to-noise ratio, and ISI correlation could minimize misplacement in the medial direction. The power of spikes in mSNr and VTA were significantly different in the high beta band (20–30 Hz), which could be an additional electrophysiological marker to distinguish mSNr and VTA in the frequency domain. Extracellular recording features that differed between mSNr and lSNr in the time domain were spike amplitude and ISI correlation. The difference in amplitude may be due to the density of neurons, thereby reducing the distance between the electrode tip and a nearby neuron. A difference in ISI correlation indicates a difference in firing pattern between the mSNr and lSNr. Interestingly, no changes in firing rate were observed between the mSNr and lSNr despite changes in firing pattern. Taken together, these results support a model of heterogeneity within the SNr due to differences not in in firing rate, but rather in firing pattern.

We speculate that differences in neuronal type may explain the lack of single-unit activity in SNc, but some in the VTA, even though electrode tracks did penetrate the SNc prior to passing through the mSNr and the lSNr. Dopaminergic neurons are highly heterogeneous and not always spontaneously active both *in vivo* and *ex vivo* (Dai and Tepper, 1998; Marinelli and McCutcheon, 2014). The VTA is composed of dopaminergic and GABAergic neurons (Merrill et al., 2015), in comparison to the SNc comprised entirely of dopaminergic neurons. The SNr contains dopaminergic neurons and GABAergic neurons with GABAergic the predominant neuronal type (Zhou and Lee, 2011). Our finding of differential firing patterns and waveform shapes in the mSNr and the lSNr parallel previous studies showing the SNr contains distinct GABAergic subpopulations (Yung et al., 1999).

There exists a paucity of studies in the literature using SNr recordings in animal models. In healthy rats, SNr neurons fired at 19.4 ± 1.2 spikes/s, while 71% of neurons had a tonic discharge, 29% fired randomly, and 0% showed a bursting firing pattern (Breit et al., 2008). One study specifically recorded from the subregions of the SNr and isolated the GABAergic neurons (Wang et al., 2010). The mean firing rate was 21.98 ± 1.27 spikes/s in the lSNr and 23.15 ± 1.33 spikes/s in the mSNr. In the lSNr, the firing pattern distribution was 54.00% regular, 40.00% irregular, and 6.00% bursting neurons. In the mSNr, the firing pattern distribution was 58.97% regular, 33.33% irregular, and 7.70% bursting neurons. Similar to our findings, no differences were observed in firing rate between the mSNr and the lSNr. In contrast to our results, however, no significant difference was found in neuronal firing patterns between the mSNr and the lSNr in healthy rats (Wang et al., 2010). These contradictory results may be explained by differences in the sex of the rats, the type of anesthesia delivered, spike sorting methodology, standard for excluding data points, and the features used to classify firing pattern. It is important that future studies reporting MERs in the SNr include the medial/lateral atlas coordinates used to allow investigators to properly interpret and reproduce the results.

In summary, changes in spike amplitude and firing pattern may be useful measures for the analysis of MERs to more reliably locate the SNr and its subregions. These findings suggest that mapping MERs to electrode position using the combined fluorescently dyed microelectrode and immunohistochemistry technique described can facilitate targeting of other deep brain targets, rather than relying on stereotaxic atlas coordinates *per se*. We suggest that LFPs in tandem with spiking activity may further facilitate MERs to promote precise targeting of microelectrodes.

## Data Availability Statement

The datasets generated for this study are available on request to the corresponding author.

## Ethics Statement

The animal study was reviewed and approved by the Stevens Institute of Technology Institutional Animal Care and Use Committee.

## Author Contributions

HL and GM designed the research and wrote the manuscript. HL performed the research and analyzed the data.

## Conflict of Interest

The authors declare that the research was conducted in the absence of any commercial or financial relationships that could be construed as a potential conflict of interest.
