# Supplementary material for: Intraoperative Microelectrode Recordings in Substantia Nigra Pars Reticulata in Anesthetized Rats
Source: Front Neurosci. 2020 Apr 29;14:367. doi: 10.3389/fnins.2020.00367 (PMC7201294; doi:10.3389/fnins.2020.00367)
Supplement: Supplementary file 1 [file Table_1.DOCX]

Supplementary Material

# Supplementary Figures and Tables

| **Features** | **VTA (n=16)** | **mSNr (n=30)** | **lSNr (n=26)** |
| --- | --- | --- | --- |
| **Firing Rate (Hz)** | 19.71±3.65 | 16.78±2.24 | 12.11±2.02 |
| **Firing Regularity** | 1.27±0.14 | 1.19±0.11 | 1.22±0.08 |
| **ISI Variance** | 0.40±0.05 | 0.46±0.05 | 0.42±0.03 |
| **Asymmetry** | 1.26±0.04 | 1.13±0.05 | 1.17±0.03 |
| **Latency (ms)** | 0.33±0.03 | 0.38±0.04 | 0.34±0.02 |
| **Noise level (μV)** | 68.72±26.03 | 57.34±18.13 | 65.44±17.62 |
| **Half-width (ms)** | 0.18±0.01 | 0.16±0.00 | 0.18±0.00 |
| **Density (%)** | 45.63±14.29 | 27.98±5.77 | 32.25±4.76 |
| **Delta 1.5-4 Hz (dB)** | 182.45±49.52 | 154.40±23.76 | 101.40±9.80 |
| **Theta 4-10 Hz (dB)** | 459.27±149.29 | 415.61±50.55 | 324.65±26.87 |
| **Beta 10-20 Hz (dB)** | 1018.00±200.16 | 857.13±86.86 | 785.61±65.98 |
| **Gamma 40-50 Hz (dB)** | 1611.10±238.74 | 1480.80±179.13 | 1125.50±147.18 |
| **Gamma 50-60 Hz (dB)** | 1693.90±227.77 | 1667.9±268.73 | 1275.10±194.38 |
| **Gamma 60-70 Hz (dB)** | 1699.50±229.54 | 1674.60±245.08 | 1274.40±233.96 |
| **Gamma 70-80 Hz (dB)** | 1608.00±237.13 | 1512.10±204.35 | 1352.60±290.82 |

**Supplementary Table 1.** **Summary of electrophysiological features in VTA, mSNr and lSNr not statistically significantly different.** Mean±SD are shown.
